# Attitudes towards poverty, organizations, ethics and morals: Israeli social workers’ shared decision making

**DOI:** 10.1111/hex.12472

**Published:** 2016-06-07

**Authors:** Lia Levin, Talia Schwartz‐Tayri

**Affiliations:** ^1^ Bob Shapell School of Social WorkTel Aviv UniversityTel AvivIsrael

**Keywords:** ethical behaviour, exclusion, Israel, organizations, shared decision making

## Abstract

**Objective:**

Partnerships between service users and social workers are complex in nature and can be driven by both personal and contextual circumstances. This study sought to explore the relationship between social workers’ involvement in shared decision making with service users, their attitudes towards service users in poverty, moral standards and health and social care organizations’ policies towards shared decision making.

**Methods:**

Based on the responses of 225 licensed social workers from health and social care agencies in the public, private and third sectors in Israel, path analysis was used to test a hypothesized model.

**Results:**

Structural attributions for poverty contributed to attitudes towards people who live in poverty, which led to shared decision making. Also, organizational support in shared decision making, and professional moral identity, contributed to ethical behaviour which led to shared decision making.

**Conclusion:**

The results of this analysis revealed that shared decision making may be a scion of branched roots planted in the relationship between ethics, organizations and Stigma.

## Introduction

Partnerships are intricate organisms. More often than not, it seems their formation, maintenance, monitoring and assessment can be gruelling, compounded by personal and organizations circumstances. Nevertheless, partnerships between social workers and service users (Various terms can be used to describe the populations that social workers provide assistance to. Inter alia, these include clients, patients, service users and more. Each of these terms implies a different conception regarding the status and essence of the assistance receiving position. As this article deals with power relations, we chose to utilize ‘service users’ to describe individuals in this position, based on the assumption that this term represents the most neutral, literal and accurate depiction of them) in the form of shared decision making and other collaborations are increasingly becoming integral components of service provision in the domain of health and social care.^1^

Generally speaking and for the purpose of this article, shared decision making with social service users can be defined using three relational conceptualizations. The first contains values as notional ethical backdrops for viewing service users as worthy partners in social interventions.^2^ Among others, these values include universalities such as equality or social justice, as well as particular ideas which link normative ethics with social ideologies, such as humanitarianism or the preservation or respect for human rights.^3^ Theoretically, these notions are often linked to critical or structural theories, which cope with social imbalances through symbolic or transformative interactions challenging power relations currently in status quo.^4^ The second touches on paradigmatic conventions concerning the epistemological and ontological assumptions service providers hold regarding the nature of social or individual truths, as well as their ability to communicate with service users in a way which uncovers genuine narratives and promotes dialectic change.^5^, ^6^ The third pertains to practical instillations of the combination of the former with the latter and offers willing social workers a range of partnership‐promoting methods and strategies.^7^ The frameworks in which these conceptualizations coexist are made up of social structures, cultural norms and on the most immediately relevant level, of health and social care organizations and systems.^8^ This contextual and perceptual complexity offers social workers inclined to partake in shared decision‐making processes with service users several potential opportunities to do so, albeit accompanied by a diverse range of challenges and tensions.^9^

It is plausible to say that today most scholars and practitioners working on the subject of shared decision making in social work agree upon two principle components which distinguish it from other forms of social work practice and from partnerships in other professional fields. One is the strong moral component it involves, which is nurtured by ethical principles guiding the social work profession. Several national social work codes of ethics, across different types of Western welfare states,^10^, ^11^, ^12^, ^13^ all mention the importance of shared decision making as a manifestation of values social work wishes to promote, namely respect and equality. The other is the nature of service users social workers are expected to form partnerships with. These service users often live in poverty, are unemployed or underemployed, deal with a variety of health needs, have very limited access to political power resources and bare severe stigma and social labels.^14^ For such groups, the formation of partnerships with social workers is expected to emphasize workers’ commitment to humanist doctrines and create symbolic interactions which have the ability to challenge and transform harmful power structures.

In Israel, participatory practices have rapidly become an integrated component of social work and social policy.^15^ Based on rights‐based critical notions in combination with quality control and new management administration,^16^ shared decision making is one of the major constituents of Israel's national social service reform programme.^17^ In terms of social problems, Israel's poverty rates have remained extremely high over past decades and so have peripheral unemployment rates, gaps in access to basic health and education, and cultural clashes on the basis of political, racial, religious or national backgrounds.^18^ Social services in Israel are provided by municipal social care services which are supervised, regulated and partially funded by government, as well as in hospitals, clinics and several non‐governmental initiatives andorganizations.^19^

Of course, the convergence between professionalism, moral perceptions, ethical behaviours and socially excluded populations may produce multifaceted dilemmas and a variety of approaches and reactions among practitioners,^20^ especially so in the Israeli context. Studies on the nature of complex partnerships between service users and social workers reveal the many difficulties workers face when approaching or practicing the principles of shared decision making. These difficulties stem from gaps between policies requiring the implementation of shared decision‐making processes and knowledge or time resources available to workers for the creation of such collaborations, cultural sensitivities which elicit conflicts between democratic notions and strict hierarchical family structures, social workers’ pre‐dispositions regarding accepting service users as knowledgeable partners, stigmatic assumptions portraying service users as unable to take responsibility for decisions made in interventions, fear that shared decision‐making questions social workers’ knowledge and expertise, and other factors associated with the organizational and professional milieu in which social workers intervene.^21^, ^22^, ^23^ Lack of belief in social change has also been found to contribute negatively to social workers’ inclination to partake in shared decision making with service users.^24^ Naturally, these professional challenges and attitudes bear significance for service users. Some service users report feeling stigmatized or disrespected when attempting to engage in partnerships with social workers, and often view health and social care systems as disregarding their potential contribution to shared decision‐making processes.^25^ Expecting all service users to willingly engage in any type of shared decision making suggested to them has also been described as having the potential to produce counter‐participatory practices and attitudes among professionals.^9^

In correspondence with the results of these studies, theoretical writing promotes the understanding that shared decision making is a process abundant with professional choices and uncertainties.^2^ Indeed, most models providing a typology of partnerships and collaborations between service providers and service users present them as ranging between potentially beneficial and empowering, representing equal power of decision or complete power of decision placed in the hands of service users, and being manipulative, placating and counter participatory.^8^, ^26^ A question remains regarding the relationship between social workers’ involvement in shared decision making with service users, the ethical or moral standards they uphold as most relevant to their practice, their attitudes towards the unique populations which use their services, and organizational policies concerning shared decision making. The study described hereby wished to provide a response to this conundrum, examining the links displayed between levels of shared decision making with service users, professional moral identities and their behavioural indices, attitudes towards poverty and service users who deal with it, and organizational dictations of the use of shared decision making among a sample of Israeli social workers.

Ethical behaviour and professional moral identity were examined separately in the current study, as were attitudes towards poverty and the people living in it, to distinguish social workers’ general professional outlooks from particular perceptions regarding their work with service users. Ethical behaviours, in our project, were defined as social workers’ self‐reported engagement in actions which epitomize the basic principles of Israel's social workers’ code of ethics. This was based on the assumptions that the code of ethics, released by the Israeli Association of Social Workers, provides an index of ethics‐based foundations for social workers’ practice in Israel and that it reflected professional discourse and terminology participants would be highly familiar with. Professional moral identity, on the other hand, was defined as the subjective appraisal of one's self as a moral professional. While ethics and morals are often related to each other, they are not interchangeable concepts. In social work, ethics comprise rules for beneficial, equal and just practices, while morals deal with choices and judgment employed when a conflict rises between two or more such rules in a given situation.^27^ In other words, ethical behaviour is the result of adhering to agreed upon, so‐perceived universal values, while morality deals with the ability to practice social work which is founded upon beneficial, real‐world decisions. And, while ethical behaviour can be the result of several elements directing behaviour, such as policy, professionalism or education, morality can be expected to be linked to more personal considerations and attitudes.^28^ Moral identity is individuals’ self‐perception of their position in world as moral beings. This self‐perception holds deep meaning for people's sense of worth and agency, as well as enhances feelings of belonging to highly regarded social milieus.^29^ As the present study dealt with social workers in their professional capacities, we chose to measure this construct as it manifests itself in their professional self‐identities.

Attitudes towards poverty, commonly operationalized as appraisals of the factors associated with the production and maintenance of poverty rates among a designated population,^30^ can be roughly divided into structural attributions (including formal or institutionalized exclusion or stratification, as well as low government investment in the advancement of adequate education, health or employment opportunities) and personal or individual attributions (including laziness, passiveness, lack of motivation, growing up in a ‘culture of poverty’^31^ or simply bad luck).^32^ As social workers in Israel work primarily with families and individuals living in poverty, it is plausible that they also hold specific attitudes towards them which result from personal encounters rather than social or economic ideologies regarding poverty as a social phenomenon.^33^

In terms of organizational support in shared decision making, social work in Israel is grounded in the public sector of social and health‐care provision, which is regulated by various organizational policies encouraging the use of such practices. These policies are diverse, ranging from policies which go into great detail regarding the way shared decision making is to be carried out, to policies that only provide social workers with very abstract statements promoting shared decision making. Also, while some policies are characterized by a binding legal status, others’ status is unclear and provides much room for professional discretion.^34^

**Figure 1:**
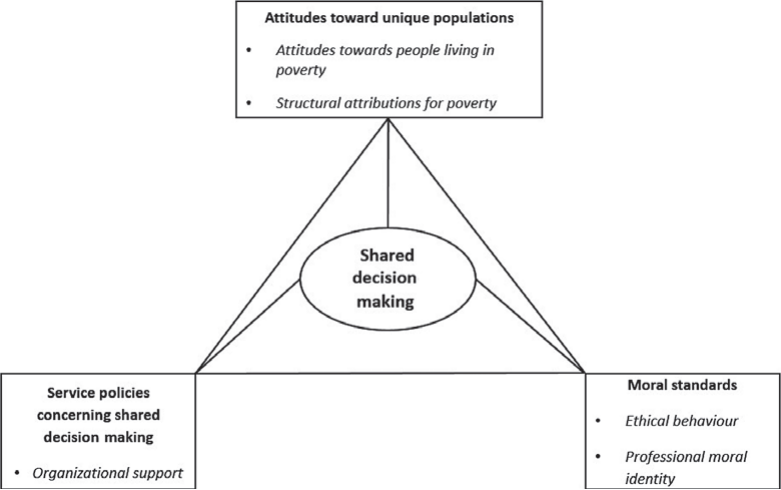
The saturated hypothetical model.

As the interaction between these factors had yet to be examined, our only hypotheses included links between them, rendered in the saturated model shown in Fig. 1.

## Methodology

#### Sample and data collection

The sample of this study included 225 licensed social workers. About 90.8% of them were women (*n* = 198). About 76.9% were employed by governmental and municipal health and social care agencies in the public sector (*n* = 176), 4.4% in the private sector (e.g. private clinics; *n* = 10) and 17% in NGOs (*n* = 39), which were mostly hospitals and Health Maintenance Organizations (HMOs). Participants’ ages ranged from 22 to 82 years, with a mean of 37.9 (SD = 11.5), and work experience ranged from a few months to 58 years, with an average of 12 (SD = 10.85). One‐way anovas showed no significant differences on all variables between public, private and third sector employees, as well as between social workers in health services and social workers in municipal and other organizations primarily concerned with social care.

Data was collected between January 2013 and May 2014, employing a cross‐sectional design. Prior to data collection, the study received the approval of Tel Aviv University's Internal Review Board. As shared decision making between social work professionals and service users is massively discussed in health and social care services in Israel and entails political as well as bureaucratic implications, to reduce bias or social desirability, data were collected via non‐official mediums. A GoogleDocs survey was used in online snowball sampling published in local professional networks, non‐official social workers’ mailing lists, Facebook communities and relevant professional discussion boards. Participation in the study was voluntary, and each participant was required to sign a virtual informed consent form before being referred to the response form itself. Participants received no direct reward for their participation.

#### Measures

*Attitudes towards people living in poverty* were measured by Cozzarelli, Wilkinson and Tagler's^30^ ‘Public Cognitive Attitudes towards the Poor’ 14‐item scale. The scale lists both positive (e.g. ‘capable’, ‘family‐oriented’) and negative (e.g. ‘criminal’, ‘dirty’) attributes. Responses were made on a 5‐point Likert‐type scale ranging from 1 = strongly disagree to 5 = strongly agree. Scores were computed by averaging responses, with higher scores indicating more positive attitudes towards people who live in poverty. Cronbach's α for this scale measured at 0.85.

*Structural attributions for poverty* were measured by 2‐item representing external attributions for poverty focused on economic or financial opportunities developed by Cozzarelli *et al*.^30^ and modified to fit Israel's political context, so that terms such as ‘federal government’ were exchanged with ‘government’, and key debates in sociopolitical discourse in the United States concerning unemployment were substituted with security expenses, an issue widely prevalent in Israel's parallel discourse. These items were as follows: ‘Discriminatory policies which exclude specific social groups from the labor force or violate their social and economic rights’; and ‘Governmental policy which prioritizes security expenses while social spending remains small‐scaled’. Responses were made on a 5‐point Likert‐type scale ranging from 1 = strongly disagree to 5 = strongly agree. Scores were computed by averaging the responses, with higher scores indicating a greater attribution of poverty to structural causes. Cronbach's α for these two items was 0.60. This reliability was lower than was to be expected in the light of the measure's original psychometric qualities, perhaps as a result of the modifications made, or due to the multifaceted relationship which exists between social divides, welfare budgeting and security considerations in Israel.

*Involvement in shared decision making* was measured by 21 items examining direct work with service users from the ‘Triadic Client Collaboration’ Inventory, converted from the principles of Levin's^8^ model of collaboration. Responses were made on a 5‐point Likert‐type scale ranging from 1 = strongly disagree to 5 = strongly agree. Examples of items comprising this inventory included the following: ‘I work together with service users on defining the goals of interventions’; ‘Partnerships with service users reflect a significant value of social work’; ‘Service users may refuse specific elements in the intervention, and such elements will not be carried out’. Scores were computed by averaging responses, with higher scores indicating greater involvement in shared decision making. Cronbach's α for this scale was 0.83.

*Organizational support* was measured using the three items representing organizational support in shared decision making in the ‘Triadic Client Collaboration’ Inventory described above. To improve internal consistency (from α = 0.62 to 0.80), one of the items was removed. Remaining items were as follows: ‘The organization I work for encourages me to work in collaboration with service users’; and ‘In the organization I work for, there are clear procedures regarding shared decision‐making between professionals and service users’. Responses were made on a 5‐point Likert‐type scale ranging from 1 = strongly disagree to 5 = strongly agree. Scores were computed by averaging responses, with higher scores indicating greater organizational support in shared decision making.

*Ethical behaviour* was measured using a 5‐item scale developed by the Authors drawing upon the Israeli social workers’ Code of ethics.^35^ Each item consisted of a core principle of the code which was converted to a question, using the intro ‘as far as it depends on me, I…’ (e.g. ‘as far as it depends on me, I aspire to increase service users’ range of opportunities’). Responses were made on a 5‐point Likert‐type scale ranging from 1 = not at all to 5 = all of the time. Scores for overall ethical behaviour were computed by averaging responses, with higher scores indicating ethical behaviour of higher standards. Cronbach's α for this measure was 0.75.

*Professional moral identity* was measured with a 12‐item Moral Identity scale developed by Aquino and Reed^29^ and modified for the current study to measure professional moral identity using the intro ‘With my clients, I am…’. Nine traits listed in the measure described moral persons (e.g. ‘caring’, ‘compassionate’, and ‘hardworking’), and three related to immoral persons (e.g. ‘selfish’) or fairly neutral individuals (‘distant’). Responses were given on a 5‐point Likert‐type scale ranging from 1 = not at all to 5 = all of the time. Scores were computed by averaging responses, with higher scores indicating a more positive professional moral identity. Cronbach's α for this measure was 0.80.

### Data analysis and findings

**Figure 2:**
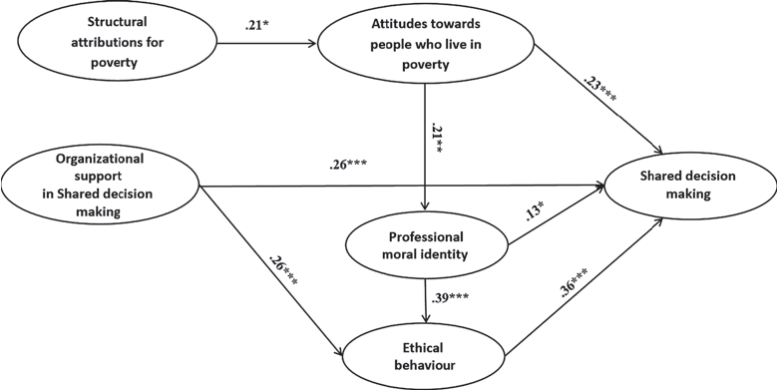
Overview of results of structural equation model analysis. Note: *P < 0.05; **P < 0.01; ***P < 0.001.

Following initial preparatory statistical examinations, structural equation modelling (SEM) was carried out using AMOS v.20.0 to incorporate mediational paths representing interactions in the model which emerged from the data. This analysis revealed good fit for observed data and highly satisfactory fit indices: χ² = 8.83, d.f. = 6, *P* = 0.18, RMSEA = 0.046, NFI = 0.95, and CFI = 0.98. Results of this path analysis are summarized in Fig. 2. As can be seen in this illustration, shared decision making was contributed to directly by attitudes towards people who live in poverty, organizational support, professional moral identity and ethical behaviour. Structural attributions for poverty contributed to shared decision making solely via attitudes towards people who live in poverty. All mediation paths were positive and significant. Both organizational support and professional moral identity contributed to shared decision making also via two additional mediation paths. Hence, the main paths found in our model were as follows: structural attributions for poverty contributed to attitudes towards people who live in poverty, which led to shared decision making; organizational support in shared decision making contributed to ethical behaviour which led to shared decision making; and professional moral identity contributed to ethical behaviour which led to shared decision making.

## Discussion

The findings of the current study shed an interesting light on the connections between structural ideologies, organizational contexts, moral identities, ethics and shared decision making as reported by social workers in Israel. As suggested by previous theoretical writing,^36^, ^37^, ^38^ social workers’ inclination to forge and maintain partnerships with service users can be explained by several factors, each tapping on a different aspect of practitioners’ working milieu and professional identity. Arguably, our findings show the utmost importance of coherence between social workers’ outlooks on poverty, organizational settings and personal attitudes, with relation to shared decision making with service users. They also demarcate that in the case of shared decision making, creeds and organizational traits translate into social workers’ approach towards specific encounters with service users and in turn lead to practical manifestations of abstract beliefs and policy guidelines.

Three major themes can be discussed to understand the model that emerged from our findings. A first set of results worth examining concerns the connection between structural attributions for poverty and shared decision making. Generally, it was unsurprising to find that attributing social problems such as poverty to power imbalances and the absence of opportunity or access to gaining social capital was associated with attitudes and practices aimed, inter alia, at increasing agency and symbolic power interactions. Both concepts deal with structural perspectives, which place the responsibility for exclusion and class hardship on harmful societal injustices, and imply the need for a social work which strives to find a balance between public interest and control on one hand, and emancipation of discriminated populations from the strife they sustain on the other.^4^, ^39^, ^40^, ^41^ In this vein, findings of the current study provide empiric evidence to what have become commonly accepted theoretical assumptions regarding the shared foundations of concepts such as poverty aware social work and participatory practices.^14^, ^42^, ^43^

Intriguingly, this link was mediated, rather than moderated or otherwise interrelated, by social workers’ attitudes towards people in poverty. This would imply that for the social workers that participated in the current study, structural perceptions bare little meaning beyond their conversion into explicit attitudes towards service users living in poverty. In other words, for them, critical ideas on discrimination and inequality‐enhancing policies contribute to their involvement in shared decision making only when attributed to specific individuals harmed by these trends. Certainly, seeing the relevance of societal ideas to the lives of service users, and the transformation of world assumptions into practical choice‐making in immediate interactions is a positive process, which may attest to consistency and multilevel professional understandings. As attitudes towards people living in poverty did not in turn contribute to general perceptions regarding structural attributions for poverty, this finding highlights the known importance^44^ of personal use of theoretical beliefs in forming real‐world reactions to professional issues. In contradiction to the findings of the current study, various studies on caregivers’ personal beliefs regarding stigmatized service user groups have shown that these increase in negativity as workers’ years of professional experience grow.^45^ An immediate implication of this would be that social work supervision, education and training capacities should invest designated time and other resources in consolidating respective and partnership worthy views of service users in poverty, beyond the exposure of workers to societal critical notions on the antecedents of poverty. Such support of these notions could assist workers in dealing with instances in which real‐world encounters challenge ideology, in ways which do not diminish their willingness to partner with service users in complex decision‐making processes.

A second major contributor to social workers’ engagement in shared decision making with service users was the extent to which workers perceived their employing organization as supportive of shared decision making. The importance of organizational cultures, norms and policies in promoting social workers’ tendency to utilize one mode of intervention over another has been documented with relation to several professional choices, including the use of policy practice,^46^ utilization of research in making practice related decisions^47^ and the engagement in evidence‐based practice.^48^ Moreover, in the current study, the primacy of organizational support of shared decision making exceeded workers’ attitudes towards poverty and service users dealing with it in explaining workers’ use of participatory practices and ideas. It is plausible to postulate that this significant, though exogenous, role organizational support played in our findings was enhanced by the fact that the vast majority of our sample consisted of social workers employed in public social care and health agencies, in which binding policies and regulations are predominant. Related to this may also be the fact that, as mentioned, shared decision making has become an increasingly prevalent component in municipal and national social work procedures in Israel.^15^ This finding underscores the relevance of organizational elements on various levels, including explicit and implicit expectations from employees, clear policies and partnership‐promoting working environments to the establishment of sustainable shared decision‐making process between social workers and their service users.

The third, most significant element in the model which ensued from our findings had to do with social workers’ professional moral identities and ethical behaviours. While these two variables were moderately linked to each other, they were clearly distinguishable in their impact on social workers’ decision making regarding partnerships with service users. Furthermore, as a conceptual unit, they represented the core mechanism of our model, acting as a hub for other connections and providing the most substantial contribution to social workers’ involvement in shared decision making.

Explanations offered for the centrality of moral identities and ethics in our model could be both conceptual and methodological. Possibly, social workers who are predominantly interested in engaging in shared decision making in ways consistent with their views on social injustices and feel that the organization which employs them echoes partnerships’ importance and reinforces their own moral identities, in turn perceive their behaviour as representing high levels of ethical standards. Furthermore, ethics have traditionally played an important role in constructing social work professional procedures. These, in terms of the importance, ethical and moral self‐perceptions hold for the social work identity, and with regard to its centrality in social work discourse and credo.^20^, ^49^, ^50^ Otherwise, it is also possible that, despite our efforts to measure distinctly different concepts, the fact that shared decision making is assumed to reflect various moral and ethical standards^2^ could imply certain affinity between shared decision making and its suggested predictors undetected as multicollinearity.

An overview of the current study and the model which emerged from its findings reveals that overall, elements dealing with direct practical work with service users living in poverty were most influential in explaining social workers’ involvement in shared decision making. This finding may correspond with two professional trends social work practice in Israel is undergoing. One is the movement towards client‐centred (also known as person‐centred) social work. Client‐centred social work is founded on principles of increasing service users’ agency and independence as well as treatment effectiveness, through offering individualized, context sensitive and personally fitted assistance.^39^ Although grounded in humanism, some link client‐centred social work to consumerist developments in social service administration, which aim at increasing service users’ satisfaction and are based on rationalizations from business‐like models of service provision.^51^ Second is a growing bottom‐up professional demand from policymakers to set clear criteria detailing service users’ rights and listing precisely which services and types of assistance they can expect to receive when approaching public health, mental health and social services. This demand is often beckoned by statements regarding the tension between social workers’ high levels of discretion and the diminishing resources allocated towards supporting their services. In this vein, arguments are made that organizations which can provide effective assistance to service users, must be founded on neutral, evidence‐based protocols, drawing upon universal ethical principles and corresponding with characteristics of the classic welfare state.^52^

In conclusion, shared decision making is a venue through which social workers can actualize their perceptions concerning deprived social groups and embed their ideological, theoretical and personal beliefs into their practice. As observed, this process is not diadic, as their encounters with service users did not produce, nor did they generate a wider understating of structural attributions for poverty. The role of ethics and morals in the context of shared decision making, as it corresponds with other elements influencing social workers’ engagement in the latter, may be instructive for understanding how to support wider uses of shared decision making with users of health and social care services. The predominant contribution of organizational support in the current study underlines the indispensable role of structured mechanisms in shaping the behaviours of social workers who wish to manifest participatory practices, across various types of organizations. When social workers’ personal attitudes and the policies that guide them converge, they share the potential of leveraging workers’ perceived ethical behaviour. This reveals the importance of enriching social workers’ professional socialization with both critical notions on stigmatized service user groups, and through policy which can lay the ground for congruous and relevant supervision, able to assist social workers in the challenging task of balancing morals, official requirements and social assumptions in the effort to enable actual shared decision making with service users. The findings of the current study also appeal to organizational policymakers who support shared decision making to produce explicit directive promoting practices associated with it. This, seeing the prominence of organizational policy among participants' considerations to engage in shared decision making, as means of promoting consistent implementation of participatory practices among social workers, and in order to convert services' credos into concrete, democratic service provision. Formulating policies which enhance unified methods of service delivery while allowing for professional judgement and flexibility in the face of the many dilemmas that rise when shared decision making takes place remain an essential challenge for policymakers.

The described study had some limitations which may be considered when reviewing its results. First, the sole reliance on self‐reported questionnaires might not reflect objective features and therefore may cause bias such as social desirability.^53^ Second, the research described was based on a convenience sample. While these sample's characteristics were similar to those of the general social work population in Israel, it cannot and should not be considered representative of this population. Third, the study examined attitudes towards poverty as a reflection of social workers’ attitudes towards excluded populations, which certainly include additional groups. Further research could replicate the suggested model using additional operationalizations which include other groups of service users. Furthermore, due to differences in policies concerning shared decision making among welfare states and to the singularity of the cultural context of Israel, the current model's validity should be examined against a variety of arenas in which social work takes place. In concordance, adaptations of concepts and methodologies to the Israeli health and social care milieu in the current study may have also influenced the model which emerged from the data collected. As may have, of course, the measures chosen for testing our variables and our choice of research design. Finally and importantly, as the current study reviewed shared decision making solely from social workers’ point of view, it certainly presents a partial account of the issues examined. Future research should be performed in order to explore the way service users perceive and react to the way shared decision making is carried out with them.
